# Supplementary material for: Effectiveness and Safety of Ayurvedic Medicines in Type 2 Diabetes Mellitus Management: A Systematic Review and Meta-Analysis
Source: Front Pharmacol. 2022 Jun 8;13:821810. doi: 10.3389/fphar.2022.821810 (PMC9213670; doi:10.3389/fphar.2022.821810)
Supplement: Supplementary file 1 [file DataSheet1.zip › Supplementary Material/Forest and Funnel Plots/Tinospora cordifolia/HbA1c.pdf]

Tinospora cordifolia - HbA1c

Study	Intervention N	Intervention Mean	Intervention SD	Comparator N	Comparator Mean	Comparator SD	Mean Diff. with 95% CI	Weight (%)
Mishra 2015	50	6.98	0.13	50	7.52	0.18	-0.54 [-0.60, -0.48]	99.43
Roy 2015	29	7.50	1.50	30	7.81	1.69	-0.31 [-1.13, 0.51]	0.57
**Overall**							-0.54 [-0.60, -0.48]	

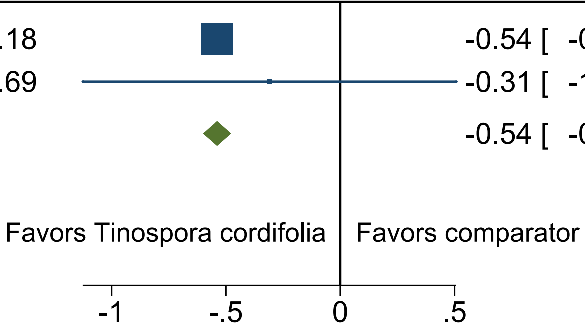

Heterogeneity: $\tau^2 = 0.00$, $I^2 = 0.00\%$, $H^2 = 1.00$

Test of $\theta_i = \theta_j$: Q(1) = 0.30, p = 0.58

Test of $\theta = 0$: z = -17.20, p = 0.00

Random-effects REML model
